# Trends in genital warts by socioeconomic status after the introduction of the national HPV vaccination program in Australia: analysis of national hospital data

**DOI:** 10.1186/s12879-016-1347-z

**Published:** 2016-02-01

**Authors:** Megan A. Smith, Bette Liu, Peter McIntyre, Robert Menzies, Aditi Dey, Karen Canfell

**Affiliations:** 1 School of Public Health, University of Sydney, Sydney, NSW 2006 Australia; 2 Prince of Wales Clinical School, UNSW Australia, Sydney, NSW 2052 Australia; 3 School of Public Health and Community Medicine, UNSW Australia, Sydney, NSW 2052 Australia; 4 The Sax Institute, Sydney, PO Box K617, Haymarket, NSW 1240 Australia; 5 National Centre for Immunisation Research and Surveillance Children’s Hospital, Westmead, Locked Mail Bag 4001, Sydney, 2145 NSW Australia; 6 Sydney Medical School, University of Sydney, Sydney, NSW 2006 Australia; 7 Present address: Cancer Research Division, Cancer Council NSW, Kings Cross NSW 1340, PO Box 572, Sydney, NSW 2011 Australia; 8 Present address: School of Public Health and Community Medicine, UNSW Australia, Sydney, NSW 2052 Australia

**Keywords:** Human papillomavirus, HPV, Vaccination, Genital warts, Impact, Socioeconomic, SES, Remoteness, Geographic, Immunization

## Abstract

**Background:**

Human papillomavirus (HPV) vaccination targeting females 12–13 years commenced in Australia in 2007, with catch-up of females 13–26 years until the end of 2009. No analyses of HPV vaccination program impact by either socioeconomic or geographic factors have been reported for Australia.

**Methods:**

Hospital admissions between July 2004-June 2011 involving a diagnosis of genital warts were obtained from a comprehensive national database. We compared sex- and age-specific admission rates in July 2006–June 2007 (pre-vaccination period) and July 2010–June 2011 (post-vaccination period) according to Index of Relative Socio-economic Disadvantage, nationally and stratified by remoteness area relating to the individual’s area of residence, using Poisson/ negative binomial models.

**Results:**

Admission rates per 100,000 population in females aged 10–19 years (predominantly vaccinated at school), reduced from 42.2 to 6.0 (rate reduction 86.7 %; 95 % CI:82.2–90.0 %) in more disadvantaged areas and from 26.8 to 4.0 (85.0 %; 95 % CI:79.7–88.9 %) in less disadvantaged areas. In females aged 20–29 years (predominantly vaccinated in the community), the decreases were from 73.9 to 26.4 (66.0 %; 95 % CI:57.7–72.6 %) and from 61.9 to 23.8 (61.6 %; 95 % CI:52.9–68.7 %) in more and less disadvantaged areas, respectively. The reductions were similar in more vs less disadvantaged areas both inside major cities (88.6 %; 95 % CI: 82.2–92.7 % vs 87.9 %; 95 % CI:82.6–91.6 % in females aged 10–19 years; 64.0 %; 95 % CI:57.0–69.9 % vs 63.8 %; 95 % CI:52.9–72.1 % for females aged 20–29 years) and outside major cities (88.8 %; 95 % CI: 83.7–92.3 % vs 85.8 %; 95 % CI:73.5–92.4 % in females aged 10–19 years; 71.1 %; 95 % CI:58.8–79.7 % vs 67.6 %; 95 % CI:48.2–79.8 % for females aged 20–29 years). Admission rates in males aged 20–29 years also reduced, by 23.0 % (95 % CI:4.8–37.8 %) and 39.4 % (95 % CI:28.9–48.3 %) in more versus less disadvantaged areas respectively.

**Conclusions:**

The relative reduction in genital warts appears similar in young females across different levels of disadvantage, including within and outside major cities, both for females predominantly vaccinated at school and in the community.

**Electronic supplementary material:**

The online version of this article (doi:10.1186/s12879-016-1347-z) contains supplementary material, which is available to authorized users.

## Background

A publicly-funded vaccination program against human papillomavirus (HPV) commenced in Australia in 2007, initially targeting 12–13 year old females and from 2013 including males aged 12–13 years. The National HPV Vaccination Program (NHVP) included catch-up programs for school-aged girls (12–17 years; delivered through schools in 2007 and 2008), young women (18–26 years; delivered in community settings until end-2009) and school-aged boys (14–15 years, delivered through schools in 2013 and 2014). The quadrivalent vaccine listed for use within the NHVP (Gardasil®, Merck&Co., Whitehouse Station, NJ USA) provides protection against HPV 16, 18, 6 and 11. HPV 16 and 18 are associated with approximately 78 % of cervical cancers in Australia [1], and HPV 6 and 11 are associated with approximately 90 % of genital warts [2].

Comparisons of vaccine uptake from the first target age-group of young females (aged 12–13 years in 2007) across different socioeconomic groups and remoteness areas of Australia estimated three-dose vaccine uptake was 71.5 % in the most disadvantaged areas versus 75.6 % in the least disadvantaged areas, and ranged from 70.1 % in remote areas to 73.6 % in major cities [3]. Less is known about the school or community-based catch-up programs. However initial studies from two Australian jurisdictions suggest uptake in young women was lower in more disadvantaged areas than in less disadvantaged areas [4, 5], and lower in inner regional areas than in either major cities or outer regional and remote areas [5].

To date there are no corresponding data on NHVP impact in Australia by socioeconomic status (SES) or geographic factors. Therefore, the aim of this study was to compare the impact of the NHVP in Australia on genital warts hospitalisations according to socioeconomic and geographic factors, both in females vaccinated at school and in the community. The indirect impact on genital warts hospitalisations in males according to socioeconomic and geographic factors was also examined.

## Material and methods

### Data sources

Data from the National Hospital Morbidity Database (NHMD), a comprehensive census of admissions to virtually all public and private hospitals in Australia, were obtained from the Australian Institute of Health and Welfare. Recorded data include information on the age, sex, geographic area of residence and Indigenous status of the individual; date of admission; primary and any contributing diagnoses; and any procedures performed. The current analysis includes all admissions with a primary or contributing diagnosis coded with the International Classification of Diseases (ICD-10) code A63.0 (anogenital warts) between 1 July 2004 and 30 June 2011, in females and males aged 10–39 years at admission. Population estimates were sourced from the Australian Bureau of Statistics website [6].

### Age groups

Admissions and population estimates were stratified by sex and classified into three age groups based on likely exposure to HPV vaccination and delivery method in females from 2007 onwards, with those 10–19 years corresponding to those predominantly offered vaccination at school, those 20-29 years predominantly offered vaccination in the community, and those 30–39 years not offered vaccination through the public program.

### Socioeconomic status (SES)

Data from the Australian Census are used to create a standard suite of indices which rank geographic areas according to different measures of socioeconomic disadvantage based on characteristics of residents within that area, for example measures of income, unemployment, occupation skill level and aspects of housing [7, 8]. Admissions and population data were classified based on the Statistical Local Area (SLA; the smallest area for which socioeconomic, health and population are available) of usual residence and that area’s corresponding Index of Relative Socioeconomic Disadvantage (IRSD) ranking, using published data [7]. Areas within IRSD deciles 1–5 were categorised as more disadvantaged, while areas within IRSD deciles 6–10 were categorised as less disadvantaged.

### Remoteness area of residence

Admissions and population data were also classified as resident inside or outside major cities, according to a published mapping between SLA and the Remoteness Area structure of the Australian Standard Geographical Classification [9], which is based on the physical road distance of the location to urban centres of different sizes [10]. As there is some correlation between the remoteness and socioeconomic index of areas (people living in more disadvantaged areas are under-represented in major urban areas but over-represented in smaller towns and remote areas) [11], this classification was used for stratifying analyses of SES.

### Statistical analyses

Consistent with previous analyses of this data [12], Poisson and negative binomial regression were used to assess overall change in admission rates between the last pre-vaccination year (July 2006–June 2007; hereafter 2006/2007) and the most recent data available (July 2010–June 2011; hereafter 2010/2011), by age group and sex. Interaction terms were used to examine whether any observed variation in admission rates since 2006/2007 differed by socioeconomic status.

A secondary analysis was performed, using admission rate ratios, comparing admission rates in each successive twelve-month period from 1 July 2007 onwards to the three-year average pre-vaccination admission rate (1 July 2004–30 June 2007). Due to the comparatively high level of missing data for remoteness area in July 2004–June 2005 (~22 %; <1 % thereafter) and SES in July 2004–June 2006 (~25 %; <1 % thereafter), the admission rate ratio was not used as the main analysis.

In order to assess whether missing data for remoteness area and SES might have biased the results, in both cases an overall analysis was done by age as in previous work [12], but restricted only to those admissions where remoteness area/ SES data were not missing.

### Additional subgroup analyses

Cervical screening: Approximately 23 % of warts admissions in females aged 10–39 years involved a procedure related to investigation or treatment of screen-detected cervical abnormalities (“screening follow-up”; Additional file 1: Table S1). Participation in cervical screening in Australia varies by SES (but relatively little by remoteness area) [13]; to exclude the possibility that variations in cervical screening behaviour between subgroups influenced the findings, we undertook a sensitivity analysis where “screening follow-up” admissions were excluded.

Men who have sex with men (MSM): As in a previous analysis [12] we examined trends in male admissions stratified according to whether the admission involved a diagnosis or procedure code associated with anal warts, or whether only non-anal sites were recorded (Additional file 1 Table S1), since anal HPV infections and HPV-related disease generally are more common in MSM [14, 15]. Admissions where the warts site could not be ascertained were excluded from this sub-analysis.

### Ethical approval statement

Ethics approval for this study was granted by the University of Sydney Human Research Ethics Committee.

## Results

Table 1 shows the distribution of admissions included in the analysis by sex, age group, SES and remoteness area.

**Table 1:** Admissions involving a diagnosis of genital warts by sex, age, socioeconomic status and area of residence, July 2004–June 2011

	More disadvantaged^a^		Less disadvantaged^a^	
**Females**				
10–19 years	947		746	
(excl. screening follow-up^b^)	784		573	
20–29 years	2,061		2,610	
(excl screening follow-up^b^)	1,594		1,955	
30–39 years	1,034		1,308	
(excl screening follow-up^b^)	834		1,020	
**Males**				
10–19 years	118		144	
20–29 years	956		1,636	
(anal site involved^c^)	429		798	
(anal site NOT involved^c^)	436		645	
30–39 years	654		1,146	
	Major cities^d^		Other areas^d^	
	More disadvantaged	Less disadvantaged	More disadvantaged	Less disadvantaged
**Females**				
10–19 years	349	563	598	183
20–29 years	984	2,171	1,077	439
30–39 years	561	1,123	473	185
**Males**				
20–29 years	506	1,402	450	234
(anal site involved^c^)	257	699	172	99
(anal site NOT involved^c^)	196	532	240	113
30–39 years	376	1,004	278	142

^a^Based on the Index of Relative Socioeconomic Disadvantage of the admitted individual’s area of residence [7, 8]. ^b^Admissions involving a procedure related to follow-up of cervical screening were excluded from this sub-analysis (see Additional file1: Table S1) ^c^Admissions were stratified according to whether the admission involved a diagnosis or treatment procedure code associated with anal warts, or whether only non-anal sites were recorded (Additional file1: Table S1); admissions where the warts site could not be ascertained were excluded from this sub-analysis ^d^Based on the Australian Standard Geographical Classification (ASGC) remoteness area of the admitted individual’s area of residence [10]. “Other areas” includes the ASGC categories Inner Regional, Outer Regional, Remote and Very Remote. In cases where the NHMD did not record remoteness area (RA) for an admission, this was assigned based on a standard Australian Bureau of Statistics (ABS) mapping for the admitted individual’s SLA [9]. In cases where that SLA contained locations with different levels of remoteness, the admission was assigned according to the standard ABS weighting for each remoteness area within the SLA [9]

### Socioeconomic status

**Fig. 1:**
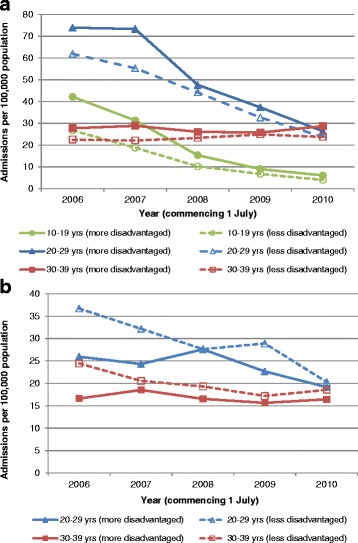
Admissions involving a diagnosis of genital warts (per 100,000 population), by age and socioeconomic status, in **a**) females and **b**) males. Males aged 10–19 years were excluded due to the small number of admissions

**Fig. 2:**
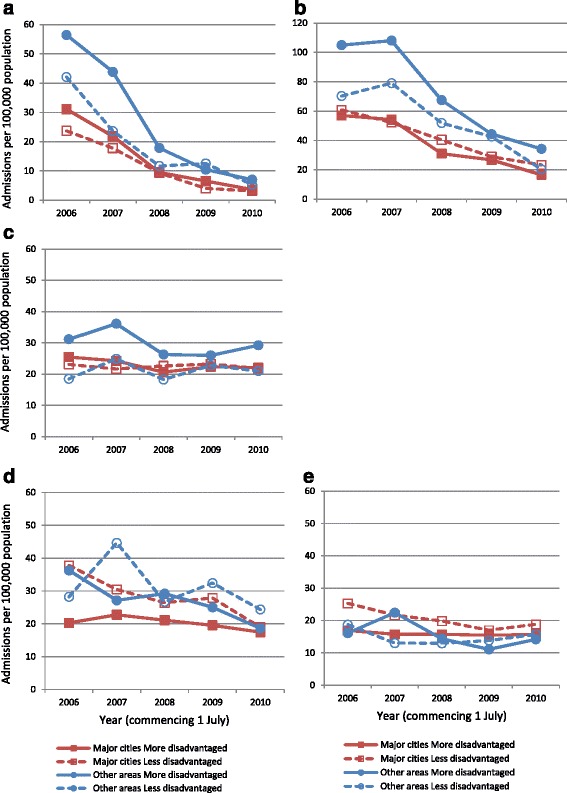
Admissions involving a diagnosis of genital warts (per 100,000 population), by age, socioeconomic status and remoteness area of residence. **a** Females 10–19 years; **b** Females 20–29 years; **c** Females 30–39 years; **d** Males 20–29 years; **e** Males 30–39 years

Annual admission rates by age, sex and SES are shown in Fig. 1 (national) and Fig. 2 (inside versus outside major cities). Significant reductions in admissions involving a diagnosis of genital warts since 2006/2007 were observed for females aged 10–19 and 20–29 years, and males aged 20–29 years, with the greatest reductions observed in females aged 10–19, followed by those 20–29 and then males 20–29 years (Table 2).

**Table 2:** Admission rates and estimated post-vaccination program reductions, by sex, age, socioeconomic status and area of residence

Group		Admission rate per 100,000^a^		Overall reduction July 2006-June 2007 to July 2010-June 2011 % (95% CI)	Interaction term P value^b^
		July 2006-June 2007	July 2010-June 2011		
**Females 10–19 years**					
National^c^	More disadvantaged	42.16	6.03	**86.7 (82.2, 90.0)**	
	Less disadvantaged	26.75	3.96	**85.0 (79.7, 88.9)**	0.57
Major cities^d^	More disadvantaged	31.13	3.62	**88.6 (82.2, 92.7)**	
	Less disadvantaged	23.73	3.16	**87.9 (82.6, 91.6)**	0.83
Other areas^d^	More disadvantaged	56.47	7.05	**88.8 (83.7, 92.3)**	
	Less disadvantaged	42.11	5.36	**85.8 (73.5, 92.4)**	0.52
**Females 20–29 years**					
National	More disadvantaged	73.93	26.39	**66.0 (57.7, 72.6)**	
	Less disadvantaged	61.87	23.82	**61.6 (52.9, 68.7)**	0.61
Major cities^d^	More disadvantaged	57.05	16.62	**64.0 (57.0, 69.9)**	
	Less disadvantaged	60.81	23.35	**63.8 (52.9, 72.1)**	0.09
Other areas^d^	More disadvantaged	105.01	34.28	**71.1 (58.8, 79.7)**	
	Less disadvantaged	70.29	20.34	**67.6 (48.2, 79.8)**	0.71
**Females 30–39 years**					
National	More disadvantaged	27.72	28.67	1.7 ( −20.4, 19.7)	
	Less disadvantaged	22.50	23.72	−9.4 ( −30.9, 8.5)	0.44
Major cities^d^	More disadvantaged	25.50	22.04	14.1 (−13.9, 35.1)	
	Less disadvantaged	23.11	21.80	1.8 (−19.4, 19.2)	0.45
Other areas^d^	More disadvantaged	31.18	29.25	17.2 (−13.5, 39.6)	
	Less disadvantaged	18.52	21.02	−5.6 (−77.2, 37.1)	0.43
**Males 20–29 years**					
National	More disadvantaged	25.95	19.17	**23.0 (4.8, 37.8)**	
	Less disadvantaged	36.69	20.28	**39.4 (28.9, 48.3)**	0.08
Major cities^d^	More disadvantaged	20.27	17.47	16.5 (−11.1, 37.3)	
	Less disadvantaged	37.81	18.96	**43.7 (33.0, 52.6)**	0.02
Other areas^d^	More disadvantaged	36.28	18.61	**42.6 (19.4, 59.1)**	
	Less disadvantaged	28.23	24.38	23.5 (−19.5, 51.0)	0.32
**Males 30–39 years**					
National	More disadvantaged	16.63	16.44	7.5 (−19.8, 28.5)	
	Less disadvantaged	24.44	18.56	**26.0 (10.1, 39.1)**	0.18
Major cities^d^	More disadvantaged	16.94	15.75	6.4 (−30.9, 33.1)	
	Less disadvantaged	25.32	18.84	**29.0 (12.7, 42.3)**	0.17
Other areas^d^	More disadvantaged	16.14	14.22	32.2 (−5.0, 56.2)	
	Less disadvantaged	18.76	15.75	13.0 (−61.8, 53.3)	0.52

Significant reductions between July 2006–June 2007 and July 2010-June 2011 in bold. ^a^Admission rate per 100,000 individuals in the population ^b^P value for whether the effect of time on admission rates (if any) differed by SES (ie P value for model interaction term) ^c^SES: socioeconomic status, based on the Index of Relative Socioeconomic Disadvantage of the admitted individual’s area of residence [7, 8] ^d^ Remoteness of admitted individual’s area of residence, based on the Australian Standard Geographical Classification [10]

In females, percentage reductions did not differ significantly between those living in more versus less disadvantaged areas, either for females aged 10–19 years at admission (predominantly vaccinated at school) or 20–29 years at admission (predominantly vaccinated in the community) (Table 2). Similar patterns were observed when admissions were stratified by residence inside versus outside major cities, with significant reductions in admissions observed across all subcategories of females aged 10–19 years and 20–29 years, but not in females 30–39 years. There was no evidence of an interaction between SES and the change in admission rates over time in females in any age group, inside or outside major cities.

In males aged 20–29 years, admission rates decreased since 2006/2007 both in more and less disadvantaged areas, with no evidence of an interaction between SES and the change in admission rates (Table 2). The findings varied for 20–29 year old males residing in major cities, however, where there was evidence of an interaction between SES and the change in admission rates (P_interaction_ = 0.02), with a significant reduction observed in less disadvantaged, but not more disadvantaged areas of major cities. There were some significant reductions in estimated admission rates for males by particular subgroupings (for example males aged 20–29 years living outside major cities in more disadvantaged areas) but there was no consistent pattern and no other evidence of interaction between SES groups.

**Fig. 3:**
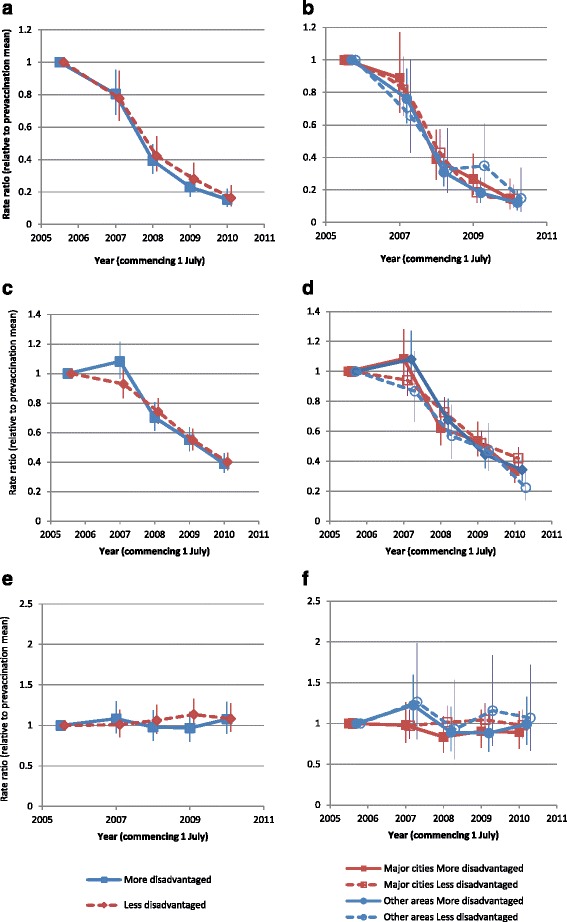
Admission rate ratio (relative to pre-vaccination mean) by age, SES and remoteness area of residence (females). **a** and **b** 10–19 years; **c** and **d** 20–29 years; **e** and **f** 30–39 years

**Fig. 4:**
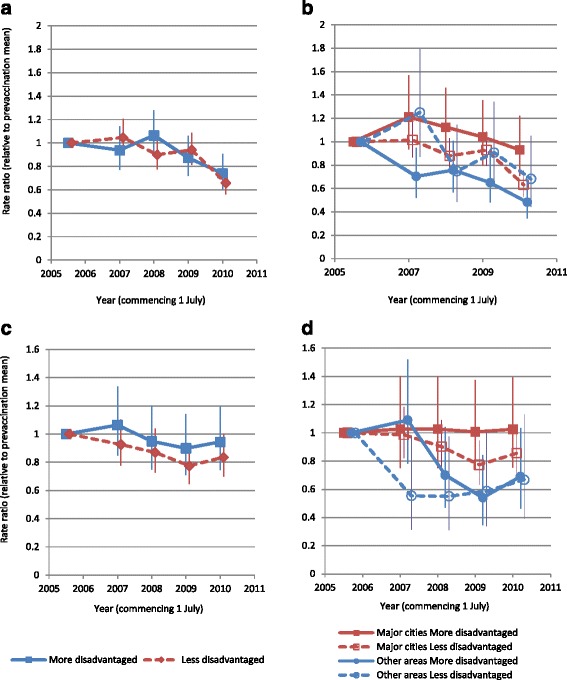
Admission rate ratio (relative to pre-vaccination mean) by age, SES and remoteness area of residence (males). **a** and **b** 20–29 years; **c** and **d** 30–39 years

The findings based on the admission rate ratio were very similar to the main analysis findings (Figs. 3 and 4). Significant reductions were observed in all subcategories for females aged 10–19 years and 20–29 years (but not in females or males aged 30–39 years). Nationally, reductions in males aged 20–29 years were significant in both more and less disadvantaged areas.

### Additional analyses

The observed patterns in females aged 10–19 and 20–29 years did not alter when admissions relating to cervical screening follow-up were excluded from the analysis. The estimated reductions within an age group did not differ significantly by SES, and the estimated reductions for each subgroup were broadly similar regardless of whether admissions relating cervical screening follow-up were included or not (Additional file 1: Table S2).

When admissions in males aged 20–29 years were stratified based on whether or not anal site was involved, the estimated reductions in admissions not involving anal site were substantial and significant, and did not differ by SES; however admissions involving anal site did differ by SES and only reduced in less disadvantaged areas (Additional file 1: Table S2). As there were apparent interactions by SES in males aged 20–29 years both in major cities and for anal warts, an additional analysis examined admissions in this age group in major cities, stratified by site. The interaction by SES for males this age residing in major cities appeared to be driven by differences in anal warts (P_interaction_ < 0.01), whereas there was a substantial and significant reduction in admissions involving only non-anal sites which did not differ by SES (Additional file 1: Table S2).

In overall analyses by age, as undertaken in previous work [12] but restricted to those admissions where SES/ remoteness area data were not missing, we found that the estimated reductions in the admission rate did not significantly differ from the original estimates based on the full set and reported in the previous analysis [12], but both the remoteness subset and the SES subset tended to result in lower point estimates of the reduction (Additional file 1: Figure S1).

## Discussion

In a previous analysis of this data, we found that the rate of genital warts admissions had decreased in school-aged girls (12–17 years), and in both young women and men (18–26 years) since the introduction of the NHVP [12]. In this further analysis, we found these reductions appear to be similar across different socioeconomic groups for teenaged-girls (10–19 years) and young women (20–29 years), and generally also for young men (20–29 years), residing both inside and outside major cities. This is the first study in Australia to examine HPV vaccine impact within subgroups based on socioeconomic or geographic factors, and one of only a small number of studies internationally to have examined vaccine impact within sociodemographic subgroups of females [12, 16]. Our findings that reductions in teenaged-females were very similar across different socioeconomic areas, including inside and outside major cities, is consistent with initial estimates of vaccine uptake from the first target age-group of young females (aged 12–13 years in 2007) which reported that uptake was relatively equal across these different groups [3]. Similar national data are not available for young women who accessed the vaccine through primary care, as the NHVP Register data is known to under-report uptake in this group [17], however data from two states suggest some potential differences. Based on data extracted from the NHVP Register for women residing in Victoria, three-dose uptake was reported as 33.4 % in the most disadvantaged areas and 38.0 % in the least disadvantaged areas [4], and in a NSW-based study of young women with a recent negative cervical screening test, self-reported uptake of one or more vaccine doses was associated with higher socioeconomic status and living outside inner regional areas [5]. We found no significant difference in the estimated reductions between women living in more versus less disadvantaged areas, although the cohorts which we examined differ slightly from those examined in the Victorian (females aged 18–26 years in 2007) and the NSW (females both aged 20–29 in 2008–2009 and 26 years or younger in mid-2007) studies. The last year of data examined here includes those aged 20–29 in July 2010-June 2011, who were potentially aged from 16–26 in mid-2007. The inclusion of slightly younger cohorts (aged 16–17 in 2007; predominantly offered vaccination at school) may potentially weaken differences in uptake between more and less disadvantaged areas if these differences were smaller in the school-based catch-up than in the community (in the youngest age group vaccinated at school, dose 1 uptake differed by only 1.2 % which is smaller than differences in the Victorian and NSW studies) [3–5]. Additionally the SES associated with the area of residence of these younger cohorts of women at the time of their hospital admission may not be the same as it was when they were vaccinated, as young women could have moved since that time. Furthermore as our results are for impact, they may not reflect exactly the same patterns as uptake, due to indirect protection effects [18]. Our finding that reductions in young men were also very similar across different socioeconomic areas, including inside and outside major cities, is also reassuring. An identified difference in the relative reduction in young men by SES within major cities appeared to be driven by differences in anal warts. No difference was observed in admissions involving only non-anal sites, which may more closely reflect indirect protection from female-only vaccination if MSM are over-represented in admissions involving anal warts.

A major strength of this study is that it uses data from a large, comprehensive and routinely-collected national dataset.

A limitation of this study is that is ecological, and vaccination status of admitted individuals is not known. However, an analysis of the same data over a longer period found the substantial reductions in admissions involving genital warts since mid-2007 were confined to young females and males, and the observed reduction did not appear to be a continuation of a pre-existing decline in either group [12]. This study is also ecological in relation to measuring socioeconomic status, since this represents the socioeconomic characteristics of the admitted individual’s area of residence, rather than the personal characteristics of the admitted individual (which were not available). However this is a widely-used approach and SLAs are the smallest area for which socioeconomic, health and population are available. Differences could also have been masked by classifying women into two SES groups rather than smaller groups such as quintiles; however an initial analysis by SES quintile also found very little difference between groups (data not shown). Another limitation was the extent of missing data on socioeconomic status prior to July 2006, and on remoteness area prior to July 2005. In order to address this, our primary analysis was restricted to use data from July 2006 on, and admission rate ratios (which used earlier years) were a secondary analysis. The findings were consistent across both the primary and secondary analyses, however, and an additional sensitivity analysis we undertook suggested that the subset we used had not biased the results (Additional file 1).

As genital warts are predominantly managed in primary care or sexual health clinics [19], these admissions data will only represent a fraction of warts cases. Admissions involving a primary diagnosis of genital warts likely represent more serious cases of warts which have not responded to earlier treatment, or where surgical treatment is not as readily available on an outpatient basis. Admissions where warts were a contributing (but not primary) diagnosis were generally for another purpose (for example treatment of cervical abnormalities in women, as previously described in the Methods). Therefore, the absolute admission rates and variation in these by SES and remoteness area could potentially relate to a combination of access and behavioural issues; for example a greater choice of treatment options outside hospitals in major cities. As the main purpose of this analysis was to examine whether the relative reduction (if any) in admission rates varied by SES, these potentially access-related differences are not likely to affect our findings unless there was a change in service usage unrelated to vaccination during the same time period; however this seems unlikely given that no changes were observed in older females.

Australia has had an organised, government-subsidised cervical screening program in place since 1991, and yet, while the program has been successful in reducing both cervical cancer incidence and mortality, socioeconomic and geographic disparities persist in cervical cancer [20]. Lower socioeconomic status is associated with lower participation in cervical screening and higher cervical cancer incidence and mortality [20]. Residing in outer regional areas of Australia is associated with higher cervical cancer mortality compared to more urban areas of Australia [20] (but not incidence, suggesting treatment variation may be a factor) [21]; while residing in remote or very remote regions is associated with both higher incidence and mortality [13]. In contrast, the uptake of school-based HPV vaccination [3] and, as reported here, its impact on genital warts, appears to have been relatively equal across socioeconomic groups including in different remoteness areas. This is potentially due to high school-participation rates [22]; the relative ease of vaccine administration (three doses) compared to repeated cervical cancer screening over many years; comparatively smaller (if any) out of pocket costs; and fewer issues of access (which for example are potentially a factor in poorer cancer survival in more remote areas) [21]. This suggests that the school-based HPV vaccination program in Australia may play an important role in reducing disparities in cervical cancer, which exist even in the context of an organised screening program and publicly-subsidised healthcare more broadly. The finding of relatively equal vaccine uptake in Australia [3, 4] is consistent with data from other countries suggesting that school-based programs result in more equitable uptake across socioeconomic strata [23–26], implying our findings on impact may also have relevance for other settings. It is therefore very important to continue to monitor both HPV vaccine uptake and impact in subgroups to ensure that the results observed to date continue. It will also be useful to assess the impact of HPV vaccination on cervical abnormalities by socioeconomic status and remoteness area to ascertain whether these findings for genital warts are also observed in cervical cancer precursors; however as records of cervical abnormalities on screening registers only include women who attend for screening and screening attendance varies by SES [13], this approach will have some limitations. Therefore monitoring of genital warts in population subgroups is likely to continue to be useful, and will additionally offer the opportunity to examine vaccine impact in subgroups of males, who have been included in the HPV vaccination program since 2013.

## Conclusions

The relative reduction in genital warts since the implementation of the NHVP in Australia appears similar in young females across different levels of disadvantage, including within and outside major cities. The impact of the program appears to have been relatively equal, both for females predominantly vaccinated at school and in the community, and also in terms of its indirect protection of males. Routinely-collected hospital admissions data on genital warts are an important source of information for ongoing monitoring of the impact of the NHVP in subgroups and in males as, while undoubtedly important in monitoring, data from cervical screening registers will not completely fulfil this role.

## Additional file

Additional file 1:
**Supplementary data. Table S1**. Summary of procedure and diagnosis codes used to define subcategories. **Table S2** Admission rates & estimated post-vaccination reductions, by sex, age, and sociodemographic features – sensitivity analyses. **Figure S1**. Admission rate ratio (admission rate in July 2010–June 2011 relative to three-year prevaccination mean (July 2004–June 2007)) estimated from the full dataset, SES subset, and remoteness area subset, by age and sex. (DOCX 34 kb)
