# Supplementary material for: Trends in genital warts by socioeconomic status after the introduction of the national HPV vaccination program in Australia: analysis of national hospital data
Source: BMC Infect Dis. 2016 Feb 1;16:52. doi: 10.1186/s12879-016-1347-z (PMC4736242; doi:10.1186/s12879-016-1347-z)
Supplement: Additional file 1: — Supplementary data. Table S1. Summary of procedure and diagnosis codes used to define subcategories. Table S2 Admission rates & estimated post-vaccination reductions, by sex, age, and sociodemographic features – sensitivity analyses. Figure S1. Admission rate ratio (admission rate in July 2010–June 2011 relative to three-year prevaccination mean (July 2004–June 2007)) estimated from the full dataset, SES subset, and remoteness area subset, by age and sex. (DOCX 34 kb) [file 12879_2016_1347_MOESM1_ESM.docx]

**Supplementary Material accompanying the manuscript:**

***Trends in genital warts by socioeconomic status after the introduction of the national HPV vaccination program in Australia: analysis of national hospital data***

Megan Smith, Bette Liu, Peter McIntyre, Robert Menzies, Aditi Dey and Karen Canfell

*Subcategory definitions for subgroup analyses*

Supplementary Table 1 - Summary of procedure and diagnosis codes used to define subcategories

| **Category** | **Procedure and diagnosis codes included ^a^** | |
| --- | --- | --- |
| ***Cervical screening follow-up related procedures ^a,b^ :*** | |  |
| Laser destruction of lesion of cervix | 35539-02 | |
| Biopsy/ cautery/ other destruction of cervical lesion | 35608 | |
| Colposcopy | 35614 | |
| Cervical cone biopsy/ others procedures/ amputation | 35618 | |
| Radical diathermy of cervix | 35646 | |
| Large loop excision of transformation zone (LLETZ) | 35647 | |
| LLETZ in conjunction with ablative treatment of additional areas | 35648 | |
| ***Warts site:*** |  | |
| Anal/ perianal/ perineal NEC | 32177, 32180; A63.01, A63.09, K62.8 | |
| Cervical warts | A63.02, N88.8 | |
| Vulval/ vaginal warts | 35507, 35508; A63.04, A63.05, N89.8, N90.8 | |
| Penile/ urethral/ scrotal warts | 36815; A63.03, A63.06, A63.07, N36.8, N48.8 | |
| *Non-anal sites only (males)* | Admissions which did not include any procedure or diagnosis codes related to anal/ perianal/ perineal warts and which did include 36815, A63.03, A63.06, A63.07, N36.8, or N48.8 | |
| *Not specified* | Admissions which did not include any of the site-specific procedure or diagnosis codes above, but did include A63.0 or A63.00. | |

*a Procedure codes correspond to Medicare item numbers and coded according to the Australian Classification of Health Interventions. Diagnosis codes correspond to ICD10-Australian Modification. NEC = not elsewhere classified. b Admissions which included none of these procedure codes were regarded as unrelated to cervical screening. Diagnosis codes were not additionally used to identify cervical screening related procedures, as diagnosis codes were not specific enough to isolate follow-up of cervical abnormalities.*

***Analyses by site in males***

When admissions in males aged 20-29 years were stratified based on whether or not anal site was involved, the estimated reductions in admissions not involving anal site were substantial and significant, and did not differ by SES (P_interaction_=0.78); however admissions involving anal site only reduced in less disadvantaged areas, and did differ by SES (P_interaction_=0.03) (Supplementary Table 2). There was an estimated 24.6% (95% CI:5.5%,39.9%) reduction in admissions involving anal site in males aged 20-29 years in less disadvantaged areas, but no reduction in more disadvantaged areas (-14.1%; 95% CI:-55.5%, 16.3%).

As there was an interaction by SES in both major cities and anal site in males this age, we further examined admissions in males aged 20-29 years residing in major cities by site (Supplementary Table 2). As for the national analysis by site, in major cities there was a significant reduction in admissions involving only non-anal sites which was substantial and did not differ by SES (P_interaction_=0.79). However, there appeared to be a strong interaction between SES and the change in rates of admissions involving anal site (P_interaction_<0.01). These were estimated to have reduced in less disadvantaged areas (28.5%; 95% CI: 9.0%, 43.9%), but not more disadvantaged areas (-35.2%; 95% CI: -102.8%, 9.9%). Therefore, it appeared that the interaction between SES and the change in rates of admissions in males aged 20-29 years residing in major cities was in practice being driven by differences in patterns of admissions for anal warts. The reductions in admissions involving only non-anal sites did not differ by SES, and these admissions are potentially more likely to represent indirect protection effects compared to those involving anal warts. Men who have sex with men (MSM) are potentially over-represented in admissions involving anal warts, and indirect protection from a female-only program is likely to be weaker in MSM ([1](#_ENREF_1)).

**Supplementary Table 2 – Admission rates & estimated post-vaccination reductions, by sex, age, and sociodemographic features – sensitivity analyses.**

| **Group** | **Admission rate per 100,000^a^** | | | **Overall reduction July 2006-June 2007 to July 2010-June 2011 (95% CI)** | **Interaction term P value^b^** |
| --- | --- | --- | --- | --- | --- |
|  | July 2006-June 2007 | | July 2010-June 2011 |  |  |
| **SENSITIVITY ANALYSES** |  | |  |  |  |
| *Females: excluding screening follow-up admissions^d^* | | | | | |
| **Females 10-19 years** |  | |  |  |  |
| More disadvantaged | 34.97 | | 5.09 | 86.4% (81.4%, 90.1%) | 0.24 |
| Less disadvantaged | 19.23 | | 3.23 | 82.0% (74.7%, 87.2%) |  |
| **Females 20-29 years** |  | |  |  |  |
| More disadvantaged | 59.69 | | 17.82 | 70.7% (59.8%, 78.6%) | 0.62 |
| Less disadvantaged | 47.09 | | 14.18 | 68.5% (57.1%, 76.9%) |  |
| *Males 20-29 years, stratified by site* |  | |  |  |  |
| **Admissions involving anal site^e^** | | | | | |
| More disadvantaged | 10.27 | | 11.96 | -14.1% (-55.5%, 16.3%) | 0.03 |
| Less disadvantaged | 17.32 | | 12.32 | 24.6% (5.5%, 39.9%) |  |
| **Admissions involving only non-anal sites^e^** | | | | | |
| More disadvantaged | 12.70 | | 6.23 | 43.4% (21.8%, 59.1%) | 0.78 |
| Less disadvantaged | 14.27 | | 6.95 | 46.7% (31.3%, 58.6%) |  |
| *Males 20-29 years residing in major cities, stratified by site* | |  |  |  |  |
| **Admissions involving anal site^e^** | | | | | |
| More disadvantaged | 8.97 | | 11.98 | -35.2% (95% CI: -102.8%, 9.9%) | <0.01 |
| Less disadvantaged | 18.33 | | 12.09 | 28.5% (95% CI: 9.0%, 43.9%) |  |
| **Admissions involving only non-anal sites^e^** | | | | | |
| More disadvantaged | 8.97 | | 4.74 | 46.3% (95% CI: 14.7%, 66.2%) | 0.79 |
| Less disadvantaged | 13.86 | | 5.98 | 50.1% (95% CI: 34.0%, 62.3%) |  |

a Admission rate per 100,000 individuals in the population b P value for whether the effect of time on admission rates (if any) differed by SES or RA (ie for model interaction term) c SES: socioeconomic status, based on the Index of Relative Socioeconomic Disadvantage of the admitted individual’s area of residence ([2](#_ENREF_2), [3](#_ENREF_3)) d Admissions involving a procedure related to follow-up of cervical screening were excluded from this sub-analysis (see Supplementary Table 1) e Admissions were stratified according to whether the admission involved a diagnosis or treatment procedure code associated with anal warts, or whether only non-anal sites were recorded (Supplementary Table 1); admissions where the warts site could not be ascertained were excluded from this sub-analysis.

***Sensitivity analysis – exploration of the effect of missing data***

There was a comparatively high level of missing data for remoteness area in July 2004-June 2005 (~22%; <1% thereafter) and SES in July 2004-June 2006 (~25%; <1% thereafter). Data from these years were not used in the main analysis, which compared admission rates in July 2006-June2007 to those in subsequent years by fitting Poisson/ negative binomial models; however they were used in the secondary analysis, involving admission rate ratios, which compared admission rates in each successive twelve-month period from 1 July 2007 on to the three-year average pre-vaccination admission rate (1 July 2004-30 June 2007). In order to assess whether the missing data for remoteness area and SES in these pre-vaccination years might have biased the results, in both cases an overall analysis was done by age as in previous work ([4](#_ENREF_4)), but restricted only to those admissions where remoteness area/ SES data were not missing. The results were compared with those from the original analysis of the full dataset ([4](#_ENREF_4)), in order to ascertain if i) the estimated admission rate ratios for July 2010-June 2011 differ significantly from those originally estimated; or ii) the estimated admission rate ratio for July 2010-June 2011 is lower than originally estimated (suggesting the change may be overestimated in the subset used for this analysis).

The results are shown in Supplementary Figure 1. The estimated admission rate ratios did not significantly differ from the original estimates based on the full set and reported in the previous analysis (16); however both the remoteness subset and the SES subset tended to result in higher point estimates of the admission rate ratios (ie lower point estimates of the *reduction*). We concluded that these subsets would not be expected to produce substantially different or biased estimates of the admission rate ratio compared to the full dataset, and if anything they may results in conservative (under)estimates of any post-vaccination program reduction in admission rates.

**Supplementary Figure 1 – Admission rate ratio (admission rate in July 2010- June 2011 relative to three-year prevaccination mean (July 2004-June 2007)) estimated from the full dataset, SES subset, and remoteness area subset, by age and sex**

*Bars represent 95% confidence interval. Original analysis of full dataset as reported in (*[*4*](#_ENREF_4)*)*

**References**

1. Donovan B, Franklin N, Guy R, Grulich AE, Regan DG, Ali H, et al. Quadrivalent human papillomavirus vaccination and trends in genital warts in Australia: analysis of national sentinel surveillance data. Lancet Infect Dis. 2011;11(1):39-44.

2. Australian Bureau of Statistics**.** Socio-Economic Indexes for Areas (SEIFA). ABS Catalogue no. 2039.0.55.001. Canberra; 2008.

3. Australian Bureau of Statistics**.** Socio-Economic Indexes for Areas (SEIFA). ABS Catalogue no. 2033.0.55.001. Canberra; 2013.

4. Smith MA, Liu B, McIntyre P, Menzies R, Dey A, Canfell K**.** Fall in genital warts diagnoses in the general and Indigenous Australian population following a national HPV vaccination program: analysis of routinely collected national hospital data. J Infect Dis. 2015;211(1):91-9.
